# The Life Crafting Scale: Development and Validation of a Multi-Dimensional Meaning-Making Measure

**DOI:** 10.3389/fpsyg.2022.795686

**Published:** 2022-03-07

**Authors:** Shi Chen, Leander van der Meij, Llewellyn E. van Zyl, Evangelia Demerouti

**Affiliations:** ^1^Human Performance Management Group, Department of Industrial Engineering and Innovation Sciences, University of Eindhoven, Eindhoven, Netherlands; ^2^Optentia Research Focus Area, North-West University (VTC), Vanderbijlpark, South Africa; ^3^Department of Human Resource Management, University of Twente, Enschede, Netherlands; ^4^Department of Social Psychology, Institut für Psychologie, Goethe University, Frankfurt am Main, Germany; ^5^Department of Industrial Psychology and People Management, University of Johannesburg, Johannesburg, South Africa

**Keywords:** life crafting, seeking challenges, seeking resources, cognitive crafting, meaning in life, wellbeing, scale development, meaning making

## Abstract

Finding meaning in our lives is a central tenet to the human experience and a core contributor to mental health. Individuals tend to actively seek the sources of meaning in their lives or consciously enact efforts to create or “craft” meaning in different life domains. These overall “Life Crafting” behaviors refer to the conscious efforts individuals exert to create meaning in their lives through (a) cognitively (re-)framing how they view life, (b) seeking social support systems to manage life challenges, and (c) actively seeking challenges to facilitate personal growth. Specifically, these behaviors are actioned to better align life goals, personal needs, values, and capabilities. However, no psychological assessment instrument currently exists to measure overall life crafting. As such, the purpose of this paper was twofold: to conceptualize life crafting and to develop, validate and evaluate a robust measure of overall life crafting. A mixed-method, multi-study research design was employed. *First*, nine participants were interviewed to determine the methods or techniques used to craft meaningful life experiences. These methods/techniques were used as indicators to create an initial item pool which was then reviewed by a panel of experts to ensure face validity. *Second*, in Study 1, the factorial structure of the instrument was explored by gathering data from a convenience sample (*N* = 331), with the results showing support for a three-factor structure of life crafting, consisting of (a) cognitive crafting, (b) seeking social support, and (c) seeking challenges. *Finally*, in Study 2 (*N* = 362), the aim was to confirm the factorial structure of the Life Crafting scale and to determine its level of internal consistency, partial measurement invariance across genders, and criterion validity [meaning in life (β = 0.91), mental health (β = 0.91), work engagement (β = 0.54), and job burnout (β = −0.42)]. The results supported a second-order factorial model of Life Crafting, which comprised of three first-order factors (cognitive crafting, seeking social support, and seeking challenges). Therefore, the Life Crafting Scale can be used as a valid and reliable instrument to measure- and track the effectiveness of life crafting interventions.

## Introduction

The COVID-19 pandemic has fundamentally changed the way individuals view life and approach work (Frenzel et al., 2022). In the absence of validated treatment strategies or vaccines, governments across the globe opted to introduce a series of non-pharmaceutical interventions (NPIs) to manage the spread of the disease (Chowdhury et al., 2020). These NPIs aimed to control the transmissibility of the disease through social distancing, case-based isolation strategies, quarantine, and community containment procedures (Frenzel et al., 2022). These NPIs led to ever-increasing restrictions on personal freedoms ranging from (inter) national travel bans, large-scale business/school/university closures, limitations on daily social engagements, and instructions to work-from-home (Van Zyl, 2021). This, in turn, resulted in large-scale layoffs, a major decline in the global economy, and radical changes in individuals’ daily activity patterns (Frenzel et al., 2022). In a relatively short period, most (if not all) individuals experienced drastic changes in how they worked, studied, shopped, and connected to others which significantly impacted their mental health and wellbeing (de Jong et al., 2020; Van Zyl, 2021). de Jong et al. (2020) argued that as loneliness and boredom set in during the initial stages of the pandemic, it led to an increase in depression, general mental health issues, and irrational decision making, which in turn increased social monitoring and eroded social bonds. In order to cope with the adverse effects of these radical changes, Lin (2021) argued that individuals started to alter the meaning they attach to and derive from these life events. Understanding the meaning or purpose of these radical changes and how individual actions, such as wearing a face mask, may contribute to the greater good, may help buffer against the negative effects these NPIs have on individuals’ mental health (Lin, 2021). Searching for or creating/crafting meaning is, therefore, an essential personal resource that individuals can employ to help cope with or make sense of the misery caused during the COVID-19 pandemic (de Jong et al., 2020; Lin, 2021).

Therefore, having a sense of purpose and meaning in our lives is a central tenet to the human experience and a core contributor to enhancing or maintaining mental health or wellbeing during times of extreme uncertainty (van Zyl et al., 2020a). Meaning, defined as “the sense that people make of their existence and having an overarching life purpose they pursue” (Steger et al., 2014, p. 27), has shown to be a critical element for better functioning in almost every life domain ranging from home to work (Steger, 2009, 2013). When individuals are actively engaging in activities which they deem to be meaningful, they are more likely to be happier (Steger, 2019), and physically healthier (Czekierda et al., 2017) as well as less likely to be depressed, stressed, or anxious (Steger et al., 2014; van Zyl et al., 2020a). When individuals are facilitated to discover what truly matters to them and are provided with the flexibility to pursue these life goals/aspirations, they show less psychopathology and show more organizational citizenship behavior, work engagement, job satisfaction, and even perform better at work (Maharaj and Schlechter, 2007; Van Zyl et al., 2010; David and Iliescu, 2020). Research has also shown that having a sense of meaning or purpose during the COVID-19 pandemic is associated with increased levels of life satisfaction, more pro-social behaviors, less psychological distress, and lower levels of negative affect over time (Lin, 2021). Further, when controlling for the presence of meaning, individuals’ perceptions of the outbreak and the adverse effects of self-quarantine did not affect individuals’ wellbeing (Lin, 2021). Given its importance, it is not surprising that practitioners, researchers, and organizations have become interested in finding practical ways to aid individuals to cultivate meaning in their lives (van Zyl et al., 2020a,b).

Jacob and Steger (2021) argued that individuals could cultivate meaning through either (a) identifying the sources of meaning in one’s life and aiding individuals to actively pursue activities aligned to such or (b) aiding individuals to craft meaning in various life domains. A considerable amount of attention has been placed on aiding individuals in identifying the sources of meaning in their lives ranging from meaning-centered therapy and positive psychology coaching to self-help activities such as photo-ethnography (c.f., Steger et al., 2014; van Zyl et al., 2020a; Richter et al., 2021). These approaches are designed to help individuals find activities which they deem to be meaningful and are facilitated to pursue these more actively to help buffer against the impact radical life challenges such as the COVID-19 pandemic has on their mental health/wellbeing (Steger et al., 2014). In contrast, aiding individuals to craft meaning in specific life domains (e.g., work-, home-, leisure-, or relationships) has only recently started to gain popularity in the literature (Tims and Bakker, 2010; Demerouti et al., 2020). From this perspective, individuals are empowered not necessarily to pursue new sources of meaning, but rather to take active steps to change the characteristics of specific life domains to be better aligned to the personal needs, values, goals, and capabilities of the individual (Tims and Bakker, 2010; Demerouti et al., 2020).

Although this crafting approach to meaning-making is becoming increasingly popular within the literature, it is stringently domain-specific and negates the dynamic interaction between life domains (de Jong et al., 2020). For example, when crafting behaviors are applied to the work context (i.e., *Job Crafting*), the aim is to align the employee’s personal needs, goals, and skills to the characteristics of the job (Tims and Bakker, 2010). This individually driven work design process provides employees with a means through which to change the nature of the tasks they engage in, change the nature of interactions at work, or modify perceptions one has about the job itself (Tims and Bakker, 2010). This approach, however, ignores the impact of other life domains such as home-life (Petrou and Bakker, 2016). When individuals are unable to experience meaning in one domain of their lives, they are likely to pursue activities in other domains such as the home environment-, leisure, or relationships to compensate (Petrou and Bakker, 2016; Demerouti et al., 2020). As an alternative, Demerouti et al. (2020) suggested that individuals may pursue “Home Crafting” to compensate for lack of meaning at work. Still viewing crafting as primarily a work-centered activity, Demerouti et al. (2020, p. 1013) defined home crafting as efforts “employees make to balance their home demands and home resources with their personal abilities and needs in order to experience meaning and create or restore their person-environment fit.” This (home) domain-specific approach also assumes that “home crafting” is fundamentally different from the behaviors associated with Job Crafting as the application domain differs. However, Demerouti et al. (2020) argue that home crafting shares similarities with job crafting in that individual actively also “seek (home) resources,” “seek (home) challenges,” and wants to “reduce (home) demands.” Therefore, it can be seen that the overarching behavioral approaches to crafting are similar. However, how and where they are applied differs. Again, this approach negates another important life domain: “Leisure.” Petrou and Bakker (2016) argued that if meaning cannot be pursued at work, it would be pursued during leisure time or activities. From this perspective, Leisure Crafting refers to “the proactive pursuit of leisure activities targeted at goal setting, human connection, learning, and personal development” (Petrou and Bakker, 2016, p. 508) pursued during one’s off-work or leisure time. From this definition, similar elements or behaviors associated with Job and Home Crafting are apparent: reshaping the task, seeking challenges, and seeking relationships. Therefore, it is apparent that people engage in similar behaviors to craft meaning in various domains of their lives.

Given the overlap in these behaviors, we, therefore, argue that crafting behaviors should be seen as a meta-level concept that transcends the confinement to particular or specific life domains. In other words, crafting should be regarded as a process of conscious efforts individuals exert to create meaning in their lives through (a) cognitively (re-) framing how they view life, (b) seeking social support systems to manage life challenges, and (c) actively seeking challenges to facilitate personal growth. Specifically, these behaviors are actioned to better align an individual’s life goals, personal needs, values, and overall capabilities. In addition, since these behaviors are directly located to an individual’s general needs, values and self-development, it may benefit all social roles individual plays *via* spillover or crossover effects from one domain to another. We call this meta-approach to meaning-making: “Life Crafting.”

As such, the present study has two goals: (1) to establish a theoretical framework for life crafting by contrasting and comparing different domain-specific crafting approaches apparent within the literature and (2) to develop and validate a positive psychological assessment measure aimed at measuring overall life crafting. Our study aims to make several contributions. First, contributing to positive psychology literature, we plan to explore how people create meaning in life by establishing the construct of life crafting. Second, we expand job crafting to the whole life domain by testing whether crafting behaviors at work are linked to crafting behaviors in life, contributing to proactivity/job crafting literature. Third, we will provide empirical evidence on the relationship between life crafting, meaning in life, and mental health. All these contributions are possible by introducing a reliable, valid, and flexible tool for empirical research on life crafting.

## The Conceptualization of Life Crafting

Life crafting has emerged as a relatively new concept in the literature, with little to no theoretical grounding underpinning its use (c.f., Schippers and Ziegler, 2019; de Jong et al., 2020; Dekker et al., 2020). Only three academic papers explicitly refer to life crafting as a specific strategy aimed to pursue meaning. First, Schippers and Ziegler (2019) viewed life crafting as a process to reflect on life and take action to increase fit among their life, values, and wishes. Second, de Jong et al. (2020) developed a four-stage theoretical life crafting intervention: discovering the values and passions, reflecting on one’s ideal life, setting specific goals and plans, and making the public commitment to the goals set. Finally, Dekker et al. (2020) argued that life crafting might enhance the individual’s goal pursuit, performance, and mental health. From these approaches, the core premise of life crafting seems to focus on proactive actions individuals take to discover their values/passions, look for challenges, and accumulate resources needed to further their personal growth and development. Although these three papers showed promise, the conceptual construction of life crafting and what it entails is severely lacking. A clear conceptual model for life crafting is needed, highlighting the concept’s theoretical foundation (and measurement).

Given that no theoretical model for life crafting exists, we turn to “crafting” in other domains (i.e., job crafting, home crafting, and leisure crafting) and use these as a reference for constructing a conceptual definition and model for life crafting. By reviewing job crafting (Wrzesniewski and Dutton, 2001; Tims and Bakker, 2010), home crafting (Demerouti et al., 2020), and leisure crafting literature (Petrou and Bakker, 2016), we attempt to find the conceptual overlap between the different crafting strategies employed in each, the definitions and structures of crafting in other domains. These are briefly summarized in Table 1. This, in turn, would act as a foundation for life crafting.

**TABLE 1:** Definitions and structures of crafting in other domains.

	Definitions	Structure
Job crafting	Job crafting is the physical and cognitive changes individuals make in the task or relational boundaries of their work (Wrzesniewski and Dutton, 2001). The actions employees take to change their levels of job demands and job resources in order to align them with their own abilities and preferences (Tims and Bakker, 2010; Tims et al., 2012).	Cognitive crafting Relational crafting Task crafting Increasing structural job resources Increasing social job resources Increasing challenging job demands Decreasing hindering job demands
Home crafting	Changes that employees make to balance their home demands and home resources with their personal abilities and needs, in order to experience meaning and create or restore their person-environment fit (Demerouti et al., 2020)	Seeking resources at home Seeking challenge at home Reducing demands at home
Leisure crafting	The proactive pursuit of leisure activities is targeted at goal setting, human connection, learning, and personal development (Petrou and Bakker, 2016).	Single dimension: leisure crafting

By contrasting and comparing the different approaches toward crafting in different domains, we found that there are clear conceptual overlap in strategies between job-, home- and leisure crafting: (a) cognitive crafting, (b) relational crafting, (c) resources crafting, (d) challenges crafting, and (e) demands crafting. There are small differences in how similar crafting behaviors manifest from these five overlapping strategies. For example, although relational crafting was an essential component of job crafting, the perspective that the researcher used to explain it is slightly different. Wrzesniewski and Dutton (2001) highlighted how people expand or restrict their social network, whereas Tims and Bakker (2010) were curious about the individual’s social resources-seeking behaviors. We, therefore, proceeded to look at the overlap and differences and derived 8 possible crafting strategies from the literature.

*Cognitive crafting* is the effort of individuals to redefine or reframe their life in such a manner that it provides more meaning. The perception of meaning in life is primarily influenced by how people think about or define their life (Beck, 1995). For example, a psychologist believing his/her work or life serves a broader purpose through mitigating mental pain or stimulating others’ flourishing. In this case, they may evaluate that their life is more meaningful because their work contributes to something conceptually larger than themselves (Wrzesniewski et al., 1997). Bruning and Campion (2018) found that cognitive job crafting was positively related to meaning. Berg et al. (2015) proposed three ways to craft individuals’ cognition: expanding perceptions (e.g., look for the holistic purpose of job), focusing perceptions (e.g., narrow the mental scope of the purpose for dislike work), and liking perceptions (e.g., connect specific tasks to adoring outcomes). Wellman and Spreitzer (2011) believed that cognitive crafting, such as thinking about best-self, could enhance scholars’ joy and meaning. Furthermore, we reviewed the items from the job crafting scales (Slemp and Vella-Brodrick, 2013; Bindl et al., 2019), and conclude that two overall cognitive crafting strategies can be distilled: positive thinking (e.g., proactively looking for the positive aspect of adverse events) and transcending personal goals (e.g., think about how your life contributes to society).

*Relational crafting* refers to people seeking social support to further pursue personal or life goals. Human beings are social animals, and interaction with others plays a vital role in their daily lives (Rofcanin et al., 2019). Relationship with others is one of the most important sources of meaning (Steger, 2009; Sacco et al., 2014). Individuals may actively choose to spend more time with preferred people and seek assistance when encountering difficulties (Laurence, 2010). Prior studies found that relational crafting can increase meaningfulness, extra-role performance, work engagement and decrease job boredom (Harju et al., 2016; Tims et al., 2016). Berg et al. (2015) proposed three ways to craft individuals’ cognition: building relationships (e.g., increasing the amount of interaction), reframing relationships (e.g., thinking about their social environment in different ways), and adapting relationships (e.g., assisting others). Further, we reviewed the items from job crafting (Tims et al., 2012; Slemp and Vella-Brodrick, 2013; Bruning and Campion, 2018; Bindl et al., 2019), and the literature about networking (Porter and Woo, 2015; Wolff and Spurk, 2020). From this review we concluded that three relational crafting strategies are relevant: crafting strategies are relevant: creating new relationships (e.g., try to meet new people), optimizing current relationships (e.g., improving the quality of my interactions with people), and utilizing social resources (e.g., seeking support from family when feeling down).

*Resources crafting* aims to achieve life goals and fulfill life’s potential by increasing or optimizing available resources. For example, individuals could look for more autonomy and seek more technological competence. Resources refer to the life circumstances people value in the pursuit of meaningful goals (Hobfoll, 1989), such as the opportunity for development and autonomy. Demerouti et al. (2001) proposed that resources in the work domain could facilitate the achievement of work goals, reduce job demands and the associated physiological and psychological costs, and stimulate personal growth and development. It can also promote essential outcomes, such as job performance (Demerouti et al., 2010) and wellbeing (Nielsen et al., 2017). Moreover, previous studies found that resources are an essential source of positive meaning (Clausen and Borg, 2011; Sacco et al., 2014). Thus, we argue that seeking life resources is a valuable crafting strategy for individuals to increase meaningful life experiences.

*Challenge crafting* is the proactive behavior that aims to help individuals experience personal growth, achievement, and accomplishment. Examples are working hard on challenging activities and seeking a new challenge in life. Lepine et al. (2005) claimed that not all job demands are related to adverse outcomes. Some types of job demands may lead to positive results, such as personal growth and positive emotions. They coined those job demands as challenging demands. Challenging demands can be viewed as a barrier in life that can be overcome with effort. If people do so, they may experience a sense of personal accomplishment. Previous studies have shown that seeking challenges increases work engagement (Petrou et al., 2012), academic performance (Ingusci et al., 2020), and job performance (Petrou et al., 2015). We argue that seeking challenging life demands will also promote a positive self-image and create meaning in life.

*Demands crafting* aims to reduce hindering life demands and avoid excessive resource loss more effectively. Examples are avoiding intense mental work and using one’s strengths to achieve life goals (Richter et al., 2021). Demands typically cost resources, but unlike the challenge demands we mentioned above, some demands will decrease individuals’ motivation and engagement (Lepine et al., 2005; Tims et al., 2012). Once this type of demand exceeds one’s capability, it will deplete mental health and wellbeing (Tims and Bakker, 2010). Although life demands cannot be avoided, we can interpret and deal with them positively or effectively, such as simplifying the work processes to make them more efficient (Demerouti and Peeters, 2018). This strategy may free people from intensive life demands and avoid stress and burnout. Notably, in previous studies, demands crafting sometimes had a negative or no relationship with other dimensions (Tims et al., 2012; Lichtenthaler and Fischbach, 2019). It may be because demands crafting was driven by avoiding motivation (Petrou et al., 2012).

Overall, the eight dimensions we included from the literature were: positive thinking, transcending personal goals, creating new relationships, optimizing current relationships, utilizing social resources, resources crafting, challenges crafting, and demands crafting.

### Nomological Network of Life Crafting

Life crafting can be embedded in the broader meaning-making literature based on several key attributes. First, life crafting is a general type of crafting, so it should be similar to crafting in other life domains. Second, life crafting highlights how people self-initiate and deliberately create meaning in life, which might overlap with proactive behaviors. Third, life crafting happens not only after adverse life events but also throughout everyday life when people proactively try to create meaning in life. Therefore, to further clarify the concept of life crafting, we will compare life crafting to job crafting, proactive personality and coping, and meaning-making theory (i.e., Meaning Maintenance Model and Global-situational Meaning-making Model).

#### Life Crafting and Job Crafting

Job crafting can be interpreted as life crafting applied to one specific domain in life. In the literature, job crafting refers to the actions employees proactively take to make their own job more meaningful, engaging, and satisfying (Wrzesniewski and Dutton, 2001; Tims and Bakker, 2010; Wrzesniewski et al., 2013). Wrzesniewski et al. (2013) proposed that job crafting is an effective strategy that helps individuals gain positive work meaning from specific sources, such as themself, their social circumstance, work context, and spirituality. Tims et al. (2016) also found that job crafting could increase meaningfulness by promoting person-job fit. Compared to job crafting, we argue that life crafting is a more general and holistic concept because life meaning could be drawn from multiple sources (Steger and Dik, 2009), instead of from a single source (e.g., the work domain). For example, Bronfenbrenner (1992) posited that meaning in life is constructed around various domains (microsystem) and the interactions of these domains (mesosystem). Thus, we conceptualized life crafting as a domain-unspecific concept.

#### Life Crafting and Proactive Personality

The proactive personality is a behavioral disposition toward taking action or changing one’s environment (Bateman and Crant, 1993). The core premise of the proactive personality is that people, behaviors, and environment have lastingly influenced each other. Therefore, people can proactively change the environment they live in Fuller and Marler (2009). Prior studies found that people with proactive personalities were more likely to attach great value to their job and precept high-level work meaning (Akgunduz et al., 2018, 2020). In line with proactive personality, life crafting emphasizes people’s initiative to change or shape their external environment. For instance, how people proactively change their environment to make it more challenging and appealing. As such, we argue that life crafting also includes a cognitive component, whereby individuals can actively change their views about life. In addition, compared to proactive personality, life crafting, as a behavior, is easier to emerge, change or enhance. Therefore, individuals could always look for the chance to craft their daily life.

#### Life Crafting and Proactive Coping

Proactive coping refers to individuals building up available resources in order to achieve challenging goals and personal growth (Schwarzer and Knoll, 2003). The basic proposition of proactive coping is that people view demands as a challenge to promotion instead of threatening resource loss (Hambrick and McCord, 2010). Greenglass and Fiksenbaum (2009) found that proactive coping could improve positive affect and psychological functioning. Similar to life crafting, proactive coping also stresses individuals’ initiative to control the situation and to seek challenges. However, the ultimate purpose of proactive coping is to handle a situation successfully or transform the potential threatens into opportunities, whereas life crafting aims to increase an individual’s positive meaning in life.

#### Life Crafting and Reactive Meaning-Making Theory

The mechanisms of meaning-making have been studied for decades. Two prevalent theories in the field were the Meaning Maintenance Model (Heine et al., 2006) and the Global-situational Meaning-making Model (Park, 2010). The Meaning Maintenance Model’s core assumption is that people tend to reaffirm alternative frameworks while experiencing meaningless or meaningful disruption. In comparison, the Global-situational Meaning-making model is used to explain how individuals’ global meaning (e.g., beliefs) interact with situational meaning (e.g., a meaningless context). They were devoted to exploring how people respond and recover from meaningless situations or mental trauma. Conversely, life crafting emphasizes people’s initiative or proactive efforts to search for meaning, it assumes that motivation for living worth will lastingly force people to pursue a better and meaningful life, instead of just when bad things happen. Moreover, the former two meaning-making theories mainly underlined reflective- or cognitive exercises. However, life crafting provided a practice-friendly framework that values action than exposed facto reflection in traumatic events. This distinguishes life crafting from other recover-oriental meaning-making theories.

#### Consequences of Life Crafting

We propose that life crafting leads to many positive outcomes for individuals. Firstly, life crafting is a strategy people use to increase meaning in life. Thus, more life crafting should be related to more meaning in life. Secondly, life crafting may lead to higher levels of mental health. Crafting is driven by individuals’ own needs (De Bloom et al., 2020). Therefore, people who craft their lives more are also more likely to experience life satisfaction and positive affect. This reasoning is in line with Dekker et al. (2020) view that life crafting is an essential strategy to improve and maintain overall mental health. Thirdly, we propose that life crafting is positively related to work-related variables (i.e., work engagement and job burnout). On the one hand, life crafting has similar effects or relationships to job crafting in the work domain. Ample studies have shown that job crafting will enhance work engagement and reduce job burnout (Bakker and Costa, 2014; Tims et al., 2015; Harju et al., 2016). On the other hand, life crafting in the non-work domain may also enhance work engagement and reduce job burnout through crossover or compensation effects (De Bloom et al., 2020). For instance, Abdel Hadi et al. (2021) found that leisure crafting behaviors were negatively related to employees’ emotional exhaustion and mitigated the undermining effect of job and home demands on the emotional exhaustion.

## The Current Study

Given the potential benefits of life crafting, the current study aimed to conceptualize life crafting and to develop, validate and evaluate a robust measure of overall life crafting.

## Materials and Methods

### Research Approach

A mixed-method, multi-study design was employed to develop and validate the Life Crafting scale. In the preliminary study, we reviewed prior research and interviewed people to create the item pool. In study 1, we performed a cross-sectional study to explore the construct of life crafting and its underlying factors. In study 2, we ran a cross-sectional study to confirm and validate the factor structure of life crafting found in study 1.

### Preliminary Study: Item Generation

The current study developed potential items through both deductive (e.g., literature review) and inductive (e.g., interview) techniques. Firstly, we collected insights from the literature on job crafting, home crafting, leisure crafting, and meaning in life. After this, we retrieved items from the Job Crafting Scale (Bindl et al., 2019), JD-R based Job Crafting Scale (Tims et al., 2012), Role–Resource Job Crafting Measure (Bruning and Campion, 2018), and the Job Crafting Questionnaire (Slemp and Vella-Brodrick, 2013). To make such items fit the life domain, we also adopted and reframed some of them. Secondly, since all of the published theoretical work on crafting behaviors are based on Western cultures, we interviewed nine participants from China (four men and five women) to explore whether there were differences in the crafting strategies these people employ. These findings were used to supplement our initial item pool. Convenient sampling was used to select interviewees. The interviewees included a painter, a novelist, two college counselors, an HRM practitioner, and four secondary school teachers. Their ages ranged from 26 to 41, and three of them were parents. We created an interview protocol based on the literature review’s findings, and in-depth semi-structured interviews were employed. We first presented and explained our definition of life crafting. Following this, we asked participants a set of open-ended questions about their approaches to creating meaning and how they experienced this. All interviewees were interviewed in Mandarin. The first author translated the interview manuscripts into English and all authors coded the materials together in English. We used content analyses (Hsieh and Shannon, 2005) as a means to process the data with the qualitative data analysis program Nvivo11. See Table 2 for the interview questions, coded answers, and the typical illustrative quotations. Following up on a reviewer’s suggestion, we also interviewed five European people to check if these strategies were consistent with those that individuals from western cultural backgrounds exhibit. The results showed that there was considerable overlap between the original findings from the Chinese sample interviews and those from Europe.

**TABLE 2:** Examples of life crafting techniques.

Questions	Typical thoughts and behaviors	Typical illustrative quotations
Can you recall specific examples of when you sought meaning by reinterpreting or reflecting on work, family, or life events?	1. Seek support from family; 2. Seek advice from others; 3. Share my life with friends or family; 4. Expand my social network.	*“I like to expand my social network, especially meet senior leaders. Because if you have a good relationship with them, it will be easier for you to deal with the problem at work.”*
Can you recall specific examples of when you sought meaning by expanding/limiting your social network or seeking support from your social network?	1. Recognize me; 2. Think about the influence on others; 3. Find a balance between life and dream; 4. Think about the influence on others.	*“I (a teacher) recognized that my actions or words might influence others. Most of my students can solve the problems by themself. So I begin to trust my students, give them positive feedback, and mentor them.”*
Can you recall specific examples of when you sought meaning by challenging yourself or fitting you and your life?	1. Learn new skills; 2. Take control of your life; 3. Look for the chance to challenge yourself; 4. Expand hobbies; 5. Take extra works.	*“I used to be scared of public speaking. I sometimes behaved in such a way that I seemed to deserve less respect. However, as a teacher, you cannot avoid public speaking. I finally find that if I am in charge of the topic, I can control my audience. So I train myself to be more dominant in large meetings. Finally, I am not nervous anymore when I have to speak to groups.”*

In step three, we created a life crafting item pool with 64 items based on our literature review (step 1) and interviews (step 2). By speculating the content of items, the first author independently classified these 64 items into eight theoretical dimensions in Round 1. There are six items for creating new relationships, seven items for optimizing current relationships, utilizing social resources, and challenges crafting, respectively, eight items for transcending personal goals, nine items for positive thinking and resources crafting respectively, and 11 items for the demands crafting. After this the other three co-authors checked the definition and category of each dimension. In Round 3, we invited a panel of experts to review our items pool to assess content validity. The panel consisted of 5 psychologists who had specifically researched crafting behaviors or meaning in life. These five experts were asked to assess the consistency between the definitions and items on a 5-point Likert scale from 1 (not representative of the concept definition) to 5 (very representative of the concept definition). We first checked the interrater reliability by calculating Cohen’s (1960) kappa values. Cohen’s kappa values ranged from −1 to 1, and values ≤ 0 as indicating no agreement, 0.01–0.20 as none to slight, 0.21–0.40 as fair, 0.41–0.60 as moderate, 0.61–0.80 as substantial, and 0.81–1.00 as almost perfect agreement (McHugh, 2012). Based on these criteria, we removed 18 items for which Cohen’s (1960) kappa values were ≤ 0. We then computed the mean score of the experts’ grades on the remaining 46 items and kept 33 items who got a three or higher mean score. Considering the experts’ comments, we also removed four items that were regarded as redundant. Moreover, two items received a low score on representativeness (2.8). We, therefore, decided to rephrase these items as we did believe the items were relevant to life crafting. Eventually, an item pool with 31 items (eight dimensions) was established and used in the follow-up studies.

### Study 1 Exploratory Factor Analysis and Reliability

#### Methods

The purpose of study 1 is to develop and examine a generic scale that can be used to measure life crafting. An exploratory factor analysis was conducted to screen the items and explore the structure of life crafting. Additionally, we tested the reliability of the life crafting scale with Cronbach’s alpha and Composite Reliability.

#### Participants

A convenience sampling strategy was used to collect data for the study. Inclusion criteria were that participants had to be 18 years or older, English-speaking, and currently employed. Three hundred eighty-five people responded to our questionnaire, and 86% of them completed all questions. In total, 331 people participated in Study 1. See Table 3 for the participants’ gender, age, marital status, employment status, and whether they had children. Almost half of the sample were women (42.9%). The participants’ ages ranged from 18 to 71, and the average age was 27.55 (*SD* = 9.85). 50.8% of participants were single, whereas 32.6% of them were in a relationship. The majority of the participants did not have children (81.0%). 44.4% of the participants worked for an organization, and 9.4% were self-employed. The most of participants were recruited from the UK and other European countries, such as Portugal, Poland, and the Netherlands.

**TABLE 3:** Demographic and biographic characteristics.

		Study 1		Study 2		Study1 vs. Study 2
Item	Category	Frequency	Percentage (%)	Frequency	Percentage (%)	*p*-value
Gender	Male	189	57.1	188	51.9	0.01
	Female	142	42.9	173	47.8	
	Other	0	0	1	0.3	
Age (years)	18∼30	245	74.0	166	45.9	0.01
	31∼45	65	19.7	46	12.7	
	46∼	21	6.3	150	41.4	
Marital status	Single	168	50.8			
	Married or in a relationship	157	47.4			
	Divorced	5	14.8			
	Widowed	1	0.3			
Have children	Yes	63	19.0			
	No	268	81.0			
Employment status	Work for an organization/company	147	44.4	317	87.6	0.01
	Self-employed	31	9.4	37	10.2	
	Other	153	46.2	8	2.2	

#### Procedure

The participants were recruited through Prolific and electronic surveys were administered through Qualtrics. The electronic questionnaire consisted of questions relating to participants’ demographic information and the 31 life crafting items. The Ethical Review Board at the Eindhoven University of Technology approved this study. This study was registered under this code: ERB2020IEISSHI20. Inclusion criteria were that participants had to be 18 years or older, English-speaking, and employed.

#### Measures

##### Life Crafting

Participants answered each of the 31 life crafting items, stemming from the original eight dimentions, in respect to how frequently they engaged in each of the mentioned behaviours. Each item was rated on a five-point Likert scale (1 = never, 2 = sometimes, 3 = regularly, 4 = often, 5 = always). Example items were ‘I change the way I think about challenges to make myself feel more positive about them’ (positive thinking), “I think about how my life contributes to society” (transcending personal goals), “I try to meet new people” (creating new relationships), “I spend more time with people who give me energy” (optimizing current relationships), “I use my social network to more effectively achieve my life goals” (utilizing social resources), “I try to learn new things” (resources crafting), “I undertake or seek extra tasks to expand my vision” (challenges crafting), and “I structure my tasks to achieve my goals” (demands crafting).

#### Statistical Analyses

To explore the factorial structure of the life crafting scale, we performed an exploratory factor analysis (EFA) with SPSS 25.0. First, the Kaiser-Meyer-Olkin (KMO) measure and Bartlett’s sphericity test were used to determine factorability. A KMO value larger than 0.60 and a statistically significant chi-square value on Barlett’s test of sphericity would indicate that the data are factorable (Dziuban and Shirkey, 1974). Thereafter, we determined the multivariate normality of the data by reviewing the absolute ranges for skewness and kurtosis. According to George and Mallery (2019), a skewness/kurtosis range between ± 2.0 indicates that the data is relatively normally distributed.

Second, an EFA was conducted with varimax rotation to extract factors. Since there are eight factors in our hypotheses, we first extracted eight factors to check the quality of the items. We only retained factors with an eigenvalue of at least 1, and the total combined explained variance of all the retained factors was set to at least explain 50% of the overall variance (Carpenter, 2018; Youssef-Morgan et al., 2022). Furthermore, we removed those items with a factor loading and commonality smaller than 0.4 or when they loaded more than 0.4 on more than one factor (Carpenter, 2018).

Finally, Corrected item-total correlation (CITC), Cronbach’s alpha, and Composite Reliability (CR) were used to examine reliability. CITC is the correlation of the designated item with the sum of other items, and the value of CITC for each item should be above 0.3 (Field, 2013; George and Mallery, 2019). Alpha is the lower bound, and CR is the upper bound of the internal consistency. Their values should be all above 0.7 (Hair et al., 1998). We ran a confirmatory analysis to get the standardized factor loading with the first sample to calculate CR with Mplus 8.0.

### Results Study 1

#### Exploratory Factor Analysis and Reliability

Since we theorized that life crafting is a model with eight first-order factors, we first fixed the number of factors to eight and then used principal factor analysis with a varimax rotation. The eight-factor model was factorable as the KMO was 0.93, and Barlett’s test indicated sphericity. The eight factors explained 49.10% of the overall variance. After that, we removed 17 items because either their loading on one of the factors was smaller than 0.4 (11 items), their commonality was smaller than 0.4 (2 items), or when an item had a high loading on more than one factor (4 items). Following Carpenter’s (2018) suggestion, we deleted the factors that included less than three items. One two-item factor, two one-item factors, and one zero-item factor (totalling four items) were deleted based on this, resulting in a four-factor model with 16 items.

Consequently, we ran a principal axis factor analysis with varimax rotation and screened the items again. The results indicated that only three factors had an eigenvalue larger than 1. Therefore, we removed the factor that got an eigenvalue smaller than 1 (3 items). Finally, we ended up with a three-factor model with nine items. The three factors explained 53.87% of the overall variance. Results showed that meaningful factors could be extracted from the data because the KMO value was larger than 0.60 (KMO = 0.82) and a significant chi-square [χ^2^_(331)_ = 1139.81, *df* = 36, *p* < 0.001] was produced. The mean, SD, CITC, and factor loading for each item were reported in Table 4. We reported the Cronbach’s alpha, CR, and correlations among three factors in Table 5. We found that these three factors were identical to the factors cognitive crafting, relational crafting, and challenges crafting of the initial eight factors. Moreover, by inspecting the contents of the nine remaining items, we labeled the three factors: cognitive crafting, seeking social support, and seeking challenges.

**TABLE 4:** Item level descriptive statistics and factor loading.

Items	Mean	SD	Skewness	Kurtosis	CITC	Factor loading		
						CC	SSS	SC
**Cognitive crafting**								
LF 10. I think about how my life helps others	3.04	1.24	0.07	−1.09	0.63	**0.61**	0.34	0.19
LF 42. I think about how my actions positively impact my community	2.78	1.19	0.22	−0.96	0.71	**0.79**	0.18	0.27
LF 44. I think about how my life contributes to society	2.86	1.20	0.19	−1.04	0.69	**0.75**	0.10	0.25
**Seeking social support**								
LF 12. I actively ask people for advice when I encounter difficulties	3.11	1.23	0.07	−1.11	0.56	0.12	**0.68**	0.10
LF 25. I seek support from my family when I am down	2.92	1.37	0.13	−1.27	0.55	0.20	**0.61**	0.17
LF 40. I am willing to ask others for help when things become too difficult to bear	3.09	1.17	0.07	−1.12	0.63	0.11	**0.78**	0.10
**Seeking challenges**								
LF 27. I try to work hard on challenging activities	3.33	1.03	−0.04	0.13	0.58	0.10	0.13	**0.65**
LF 41. I change my activities so that they are more challenging	2.40	1.00	0.67	0.13	0.64	0.34	0.12	**0.67**
LF 43. I seek out opportunities that challenge my skills and abilities	2.95	1.11	0.10	−0.08	0.69	0.34	0.17	**0.76**

*CITC, Corrected item total correlation; λ, Standardized factor loadings; CC, cognitive crafting; SSS, seeking social support; SC, seeking challenges. Bold: Significant item loadings (p < 0.01).*

**TABLE 5:** Factor correlations and internal consistencies of life crafting.

No	Factor	CR	Cronbach’s alpha	1	2
1	Cognitive crafting	0.83	0.82	−	
2	seeking social support	0.76	0.75	0.25*	−
3	Seeking challenges	0.79	0.79	0.27*	0.19*

*p < 0.01.

### Study 2. Confirmatory Factor Analysis, Validation, and Measurement Invariance

#### Methods

In Study 2, we investigated if the three-factor structure found in study 1 could also be confirmed in a different sample. To this end, we performed a second study with confirmatory factor analysis. We compared the model fit between the three-factor model and other alternative models: the one-factor model, two-factor model, second-order factor model, and Bi factor model. Second, we examined the second-order factor model’s convergent validity and discriminant validity. There is no other life crafting scale yet, so we tested convergent validity and discriminant validity by comparing life crafting to similar concepts: job crafting (Tims et al., 2012) and proactive personality (Bateman and Crant, 1999). Furthermore, we tested measurement invariance across gender. Finally, we examined criteria validation by computing the standardized regression values among life crafting, job burnout, work engagement, meaning in life, and mental health.

#### Participants

Four hundred thirty-one employees participated in Study 2, and 78% of participants filled all questionnaires. The final sample consisted of 362 participants after we deleted unfinished responses. Almost all the participants were Dutch, and their gender, age, and employment status are summarized in Table 3. Almost half of the participants were women (47.8%), and the average age was 38.60 (*SD* = 14.14). Most of the sample was working for an organization (87.6%). The average workload of the participants was 35.81 h (*SD* = 9.13) per week. We compared the biographic characteristics between participants of Study 1 and Study 2 *via t*-test and chi-square test, (c.f. Table 3).

#### Procedure

For Study 2, participants were recruited by students who participated in a Master’s course in Performance Management at the Eindhoven University of Technology in the Netherlands. Each student recruited approximately 5 participants from their social network (e.g., parents and friends), and the students then used the collected data for their assignment. Participants had to be over the age of 18 and had to work a minimum of 3 days per week. Questionnaires were administered through Qualtrics. Because we conducted the study in the Netherlands, we also asked them to report their English level with a 7-point Likert scaling ranging from 1 (Not Sufficient) to 7 (Sufficient). Eight participants who reported an English level below three were removed from the analyses. The Ethical Review Board at the Eindhoven University of Technology approved this study, and this study was registered under this code: ERB2020IEIS20.

#### Measures

The Following Scales Were Administered in Study 2:

*Life crafting*. The life crafting scale developed in study 1 was used to measure life crafting. The nine-item scale was rated on a five-point Likert-type agreement scale ranging from 1 (Never) to 5 (Always). The scale consisted of nine items and comprised four subscales: cognitive crafting (3 items), seeking social support, and seeking challenges (3 items). Example items were “I think about how my life helps others” (cognitive crafting), “I actively ask people for advice when I encounter difficulties” (seeking social support), and “I try to work hard on challenging activities” (seeking challenges). The Cronbach’s α of the three subscales were 0.82, 0.75, and 0.79. The final set of items is presented in Appendix Table 1.

*Job crafting.* We adopted items from the daily job crafting scale (Petrou et al., 2012) to measure job crafting. The scale consisted of thirteen items and comprised four subscales: seeking job resources (5 items), seeking challenges (4 items), and reducing demands (4 items). Responses were given on a 5-point scale with 1 (Never) – 5 (Always). Example items were “I try to learn new things at work’ (seeking job resources), “I ask for more tasks if I finish my work” (seeking challenges), and “I try to ensure that my work is emotionally less intense” (reducing demands), The Cronbach’s α of the three subscales in this study were 0.85, 0.82, and 0.76, respectively.

*Meaning in Life*. The Meaning in Life Questionnaire developed by Steger et al. (2006) was used to measure meaning in life. The ten-item questionnaire is rated on a seven-point Likert-type scale ranging from 1 (absolutely untrue) to 7 (absolutely true). It measures the two components of meaning in life with five items each. Example items are “I understand my life’s meaning” (presence of meaning), and “I am always looking to find my life’s purpose” (search for meaning). The Cronbach’s α of the two subscales were both 0.93.

*Proactive personality.* The six-item short version of the Proactive Personality Scale (Bateman and Crant, 1993) was used to measure proactive personality. This 6-item short version was validated by Claes et al. (2005). Ratings were made on a 5-point scale that ranged from 1 (Totally disagree) to 5 (Totally agree). Cronbach’s α in this study was 0.79. Example items were “If I see something I don’t like, I fix it.” and “I excel at identifying opportunities.”

*Job burnout.* The Oldenburg Burnout Inventory (Demerouti et al., 2003) was used to measure job burnout. The sixteen-item scale was rated on a four-point Likert-type scale ranging from 1 (Strongly disagree) to 4 (Strongly agree). The scale consists of two components of job burnout with eight items each. Example items were “It happens more and more often that I talk about my work in a negative way” (disengagement) and “There are days when I feel tired before I arrive at work” (exhaustion). Half of the items were reversed coded. The Cronbach’s α of the two subscales in this study were 0.73 and 0.78, respectively.

*Work engagement.* The 9-item version of the Utrecht Work Engagement Scale (Schaufeli et al., 2006) was used to measure work engagement. The nine-item scale was rated on a seven-point Likert-type scale ranging from 0 (Never) to 6 (Always). It measured the three components of work engagement with three items each. Example items were “At my work, I feel bursting with energy (vigor),” “I am enthusiastic about my job” (dedication), and “I get carried away when I am working” (absorption). The Cronbach’s α of the three subscales in this study were 0.82, 0.88, and 0.67, respectively.

*Mental health*. The Mental Health Continuum-Short Form validated by Lamers et al. (2011) was used to measure mental health. The form consists of fourteen items that were derived from Midlife Development in the United States (Keyes, 2002). Respondents rated the frequency of every feeling in the past month on a 6-point Likert scale from 1 (never) to 7 (every day). It measures the three components of mental health with three items (emotional wellbeing), five items (psychological wellbeing), and six items (social wellbeing), respectively. Example items are “In the past month, how often did you feel happy,” “In the past month, how often did you feel that you liked most parts of your personality,” and “In the past month, how often did you feel that our society is becoming a better place for people.” The instrument showed to be a reliable measure in other contexts with McDonald omegas ranging from 0.76 to 0.92 on the various subscales (Van Zyl and Ten Klooster, 2022). The Cronbach’s a of the three subscales in this study was 0.89 for emotional wellbeing, 0.87 for psychological wellbeing, and 0.78 for social wellbeing.

### Statistical Analyses

First, we estimated factorial validity by conducting a confirmatory factor analysis in Mplus v 8.0 (Muthén and Muthén, 2020). The parameters were calculated through the maximum likelihood estimation method. Several fit indices, which we illustrated in Table 6, were used to evaluate model fit. We also calculated factor loadings, item-level statistics, and internal consistency to investigate the three-factor life crafting model and higher-order life crafting model in SPSS and Mplus.

**TABLE 6:** Model fit indices.

Fit indices	Cut-off criterion
***Absolute fit indices***	
Chi-Square (χ^2^)	• Lowest comparative value between measurement models
	• Non-significant chi-square (*p* > 0.01)
	• Significant difference in chi-square between models
	• For model comparison: retain model with lowest chi-square
***Approximate fit indices***	
Root-means-square error of approximation (RMSEA)	• 0.06–0.08 (marginally acceptable); 0.01–0.05 (excellent)
	• Not-significant (p > 0.01)
	• 90% Confidence interval range should not include zero
	• For model comparison: retain model where ΔRMSEA ≤ 0.015
Standardized root mean square residual (SRMR)	• 0.06–0.08 (marginally acceptable); 0.01–0.05 (excellent)
	• For model comparison: retain model where ΔSRMR ≤ 0.015
***Incremental fit indices***	
Comparative fit index (CFI)	• 0.90–0.95 (marginally acceptable fit); 0.96–0.99 (excellent)
	• For model comparison: retain model with highest CFI value (ΔCFI > 0.01)
Tucker-Lewis index (TLI)	• 0.90–0.95 (marginally acceptable fit); 0.96–0.99 (excellent)
	• For model comparison: retain model with highest TLI value (ΔTLI > 0.01)
Akaike information criterion (AIC)	• Lowest value in comparative measurement models
Bayes information criterion (BIC)	• Lowest value in comparative measurement models
Sample-size adjusted BIC (aBIC)	• Lowest value in comparative measurement models

*These indices and criteria were adapted from Wong and Wong (2020).*

Second, we investigated measurement invariance or factor equivalence across gender by computing and comparing configural- (similar factor structures), metric- (similar factor loadings), and scalar (similar intercepts) models in Mplus. Invariance was determined through a non-significant difference in chi-square, CFI (Δ < 0.01), TLI (< 0.01), RMSEA (Δ < 0.015), and SRMR (Δ < 0.015) (Cheung and Rensvold, 2002). If full invariance could not be established, partial invariance would be pursued by releasing some constraints on the various models (van de Schoot et al., 2012; Van Zyl and Ten Klooster, 2022).

Third, to investigate convergent validity, we created a structural equation model in which we regressed subfactors of job crafting on life crafting and life crafting on proactive personality. Additionally, we assessed discriminant validity following (Rönkkö and Cho, 2020) approach. We calculated the confidence intervals of the correlation between life crafting and similar concepts [i.e., seek resources, seek (job) challenges, reduce demands, and proactive personality], then investigated whether the upper limit values of confidence intervals were smaller than 0.90.

Finally, the same method to establish convergent validity was applied to establish concurrent validation. Using a structural equation model, we determined the relationship between life crafting and related theoretical variables (i.e., job burnout, work engagement, mental health, and meaning in life).

### Result Study 2

The confirmatory factor analyses, convergent validity, discriminant validity, measurement invariance, and criteria validity are reported in this section. The results are presented in the tabulated format with brief subsequent interpretations.

### Confirmatory Factor Analysis

We employed a competing measurement modeling strategy to establish the factorial validity of the life crafting scale. The following models were estimated:

a) Model 1: All items load on a single factor.
b) Model 2a: Items load on two factors: factor 1 (cognitive crafting + seeking social resources) and factor 2 (seeking challenges).
c) Model 2b: Items load on two factors: factor 1 (cognitive crafting + seeking challenges) and factor 2 (seeking social resources).
d) Model 2c: Items load on two factors: factor 1 (seeking social resources + seeking challenges) and factor 2 (cognitive crafting).
e) Model 3: Items load on three factors: cognitive crafting, seeking social resources, and seeking challenges.
f) Model 4: Second-order factor model: Items load on three factors: cognitive crafting, seeking social resources, and seeking challenges. Moreover, these three factors load on a second-order factor: life crafting.
g) Model 5: Bi factor model, items load on a general factor life crafting and three specific factors: cognitive crafting, seeking social resources, and seeking challenges.

**FIGURE 1:**
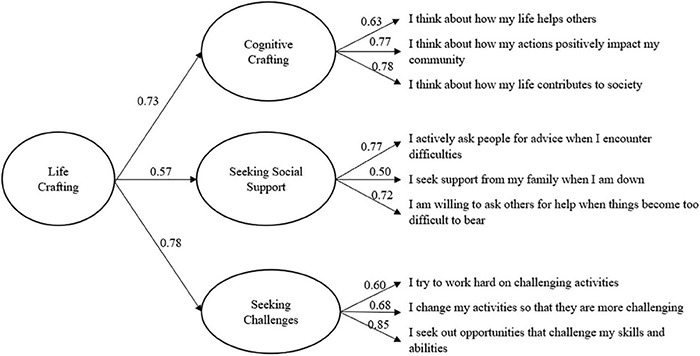
The factorial model of the Life Crafting Scale.

Table 7 presents the model fit indices for each of the six estimated models. The results showed that Model 3, Model 4, and Model 5 had a good fit and were significantly better than Model 1 and Model 2a–2c. Furthermore, Model 3 and Model 4 have identical model fits because we only have three first-level factors (Wong and Wong, 2020), and there were no statistically significant difference when compared to Model 5 (Δχ^2^ = 5.91, Δ*df* = 6, *p* = 0.43). In addition, we examined the measurement quality (Table 8) of Model 3 and Model 4. Both model 3 and 4 showed acceptable standardized factor loadings (λ > 0.40, *p* < 0.001), standard errors, and item uniqueness (0.10 > δ < 0.90, *p* < 0.001) (Asparouhov and Muthén, 2009). A defect of the measurement quality is that the AVE for Seeking social support was slightly less than 0.5. However, according to Fornell and Larcker (1981), AVE is a more conservative measure indicator than CR. If the CR value is adequate, the results should be accepted. Although model 3 and Model 5 had a good model fit and measurement quality, we argue that the second-order factor could account for the variation among the first-order factors. Therefore, we used model 4 (Second-order factor model) for the subsequent tests of measurement quality (c.f. Figure 1).

**TABLE 7:** Confirmatory factor analysis.

Model	χ^2^	*df*	χ^2^/*df*	CFI	TLI	RMSEA		SRMR	AIC	BIC	aBIC
Model 1	300.87	27	11.14	0.70	0.59	0.17	[0.151–0.185]	0.09	8777.59	8882.67	8797.01
Model 2a	169.85	26	6.53	0.84	0.78	0.12	[0.106–0.142]	0.07	8648.57	8757.54	8668.71
Model 2b	171.05	26	6.58	0.84	0.78	0.12	[0.107–0.142]	0.07	8649.78	8758.74	8669.91
Model 2c	188.46	26	7.25	0.82	0.75	0.13	[0.114–0.149]	0.08	8667.18	8776.15	8687.32
Model 3	29.67	24	1.24	0.99	0.99	0.03	[0.000–0.052]	0.02	8512.40	8629.14	8533.97
Model 4	29.67	24	1.24	0.99	0.99	0.03	[0.000–0.052]	0.02	8512.40	8629.14	8533.97
Model 5	23.76	18	1.32	0.99	0.99	0.03	[0.000–0.059]	0.02	8521.90	8662.00	8547.79

*χ^2^, Chi-square; df, degrees of freedom; TLI, Tucker-Lewis Index; CFI, Comparative Fit Index; RMSEA, Root Mean Square Error of Approximation [90%CI]; SRMR, Standardized Root Mean Square Residual; AIC, Akaike Information Criterion; BIC, Bayes Information Criterion; aBIC, Adjusted Bayes Information Criterion.*

**TABLE 8:** Item level descriptive statistics, standardized factor loadings, average value explained, and internal consistency for the second-order model.

Items	x̄	σ	Skewness	Kurtosis	CITC	λ	SE	δ	AVE	CR	α
***Cognitive crafting***									0.53	0.77	0.77
I think about how my life helps others	3.09	1.04	0.08	−0.79	0.55	0.63	0.04	0.61			
I think about how my actions positively impact my community	2.86	0.99	0.27	−0.59	0.63	0.77	0.03	0.41			
I think about how my life contributes to society	2.75	1.01	0.19	−0.85	0.60	0.78	0.03	0.39			
***Seeking social support***									0.45	0.71	0.69
I actively ask people for advice when I encounter difficulties	3.13	1.02	0.20	−0.95	0.56	0.77	0.04	0.40			
I seek support from my family when I am down	3.09	1.23	0.15	−1.07	0.42	0.50	0.05	0.75			
I am willing to ask others for help when things become too difficult to bear	3.17	1.04	0.17	−1.05	0.53	0.72	0.04	0.49			
***Seeking challenges***									0.52	0.76	0.75
I try to work hard on challenging activities	3.46	0.98	−0.22	−0.66	0.53	0.60	0.04	0.64			
I change my activities so that they are more challenging	2.54	0.88	0.42	−0.34	0.57	0.68	0.04	0.54			
I seek out opportunities that challenge my skills and abilities	3.02	0.96	0.45	−0.70	0.64	0.85	0.03	0.28			
***Life crafting***											
Cognitive crafting						0.73	0.07	0.47			
Seeking social support						0.57	0.06	0.68			
Seeking challenges						0.78	0.07	0.36			

*¯, Mean; σ, Standard deviation; CITC, Corrected item total correlation; λ, Standardized factor loadings; SE., Standard Error; δ, Item Uniqueness; AVE, average value explained; CR, Composite Reliability; α, Cronbach’s Alpha.*

### Measurement Invariance

Measurement invariance across genders (males: 188 vs. females: 173) was computed for the second-order CFA model, and the results were reported in Table 9. The results showed that all invariance models fitted the data based on the criteria mentioned in Table 6. After that, the χ^2^ difference test suggested no significant difference between the configural invariance model, the first-order metric invariance model, and the second-order metric invariance model could be found. Moreover, the difference in CFI and TLI did not exceed 0.01, RSMEA and SRMR did not exceed 0.015 for these model comparisons. Thus, we provided evidence for the configural and metric invariance.

**TABLE 9:** Measurement invariance across genders.

Model	χ^2^	*df*	CFI	TLI	RMSEA	SRMR	AIC	BIC	Model comparison	Δχ^2^	Δ CFI	Δ TLI	Δ RMSEA	Δ SRMR
M1 configural invariance	58.907	48	0.986	0.978	0.035	[0.000–0.063]	0.040	8492.916	8726.249	−	−	−	−	−	−
M2 metric invariance: first order	67.920	54	0.982	0.976	0.038	[0.000–0.063]	0.052	8489.751	8699.750	M1 vs. M2	9.01	0.004	0.002	0.003	0.012
M3 metric invariance: second order	68.623	56	0.983	0.979	0.035	[0.000–0.061]	0.053	8486.330	8688.551	M2 vs. M3	0.70	0.001	0.003	0.003	0.001
M4 scalar invariance: first order	73.279	62	0.983	0.985	0.032	[0.000–0.057]	0.057	8478.791	8657.679	M3 vs. M4	4.66	0.000	0.006	0.003	0.004
M5 scalar invariance: second order	95.830	64	0.958	0.953	0.052	[0.029–0.073]	0.075	8498.865	8669.976	M4 vs. M5	22.55*	0.025	0.028	0.020	0.018
M6 partial invariance	76.314	63	0.982	0.980	0.034	[0.000–0.059]	0.059	8479.884	8654.884	M4 vs. M6	3.04	0.001	0.005	0.002	0.002
M7 strict invariance first order	80.600	69	0.985	0.984	0.031	[0.000–0.055]	0.058	8471.886	8623.552	M6 vs. M7	4.29	0.003	0.004	0.003	0.001
m8 strict invariance Second order	82.517	71	0.985	0.985	0.030	[0.000–0.055]	0.057	8469.788	8613.676	M7 vs. M8	1.92	0.000	0.001	0.001	0.001

χ^2^, Chi-square; df, degrees of freedom; TLI, Tucker-Lewis Index; CFI, Comparative Fit Index; RMSEA, Root Mean Square Error of Approximation [90%CI]; SRMR, Standardized Root Mean Square Residual; AIC, Akaike Information Criterion; BIC, Bayes Information Criterion; aBIC, Adjusted Bayes Information Criterion. *p < 0.05.

However, the imposed scalar invariance model showed a substantial deterioration in model fit in terms of the χ^2^, CFI, TLI, RSMEA, and SRMR for the second-order factor model. For this reason, the full second-order scalar invariance had to be rejected. By checking the model comparison results, we found that the misfit between the first-order scalar model and the second-order scalar model may attribute to unequal intercepts of seeking social support. The estimated intercept of seeking social support was 3.16 in the female group, whereas the intercept in the male group was 3.01. After we freed the interception in model 6 (Partial Invariance Model), the model fit improved, and there was no difference in the χ^2^, CFI, TLI, RSMEA, and SRMR compared to model 4. Thus, partial invariance was established, and all subsequent models were based on this partial model. Finally, we set all errors in the first and second-factor levels to be equal across genders. The results showed no difference in the χ^2^, CFI, TLI, RSMEA, and SRMR. Therefore, strict invariance was established.

### Convergent Validity and Discriminant Validity

The relationships between the life crafting and similar concepts (i.e., seeking job resources, seeking job challenges, reducing job demands, and proactive personality) can be found in Table 10. The structural model of these variables showed adequate fit [χ^2^_(331)_ = 600.80, *df* = 340, χ^2^/*df* = 1.77, CFI = 0.93, TLI = 0.92, RMSEA = 0.46 (0.040, 0.052), SRMR = 0.06, AIC = 25116.47, BIC = 25482.29]. First, life crafting was directly associated with seeking job resources (β = 0.69, *p* < 0.001, *R*^2^ = 0.47) and seeking job challenges (β = 0.58, *p* < 0.001, *R*^2^ = 0.33). Second, there was no significant relationship between life crafting and reducing job demands (β = 0.06, *p* = 0.34, *R*^2^ = 0.00). Finally, proactive personality positively predicted life crafting (β = 0.46, *p* < 0.001, *R*^2^ = 0.21).

**TABLE 10:** Relationships with job crafting and proactive personality.

Regressive path	Standardized					Validity established
	β	SE	*t*-value	*p*	*R* ^2^	
Life crafting→ seek job challenges	0.578	0.051	11.447	<0.01	0.334	Yes
Life crafting→ reduce job demands	0.064	0.066	0.963	<0.01	0.004	No
Life crafting→ seek job resources	0.686	0.051	13.505	<0.01	0.470	Yes
Proactive personality → life crafting	0.457	0.057	8.035	<0.01	0.209	Yes

Table 11 presents the correlations among the three life crafting subfactors with the three job crafting scales and proactive personality. The upper limits ranged from 0.11 to 0.69. According to Rönkkö and Cho (2020), the upper limits of confidence intervals should be smaller than 0.9. Therefore, discriminant was established.

**TABLE 11:** Confidence intervals of the correlation among life crafting, three job crafting subfactors, and proactive personality.

Variable	Seek job resources	Seek job challenges	Reduce job demands	Proactive personality
Life Crafting	[0.610 0.812]	[0.463 0.668]	[−0.067 0.199]	[0.344 0.577]

### Criterion Validity

To establish criterion validity, we examined the relationship between life crafting and job burnout, work engagement, mental health, and meaning in life. The results are summarized in Table 12. We first conducted a structure model regressing life crafting on mental health and meaning in life in sample 1 (*n* = 331). The model showed adequate fit [χ^2^_(331)_ = 1090.09, *df* = 484, χ^2^/*df* = 2.25, CFI = 0.92, TLI = 0.91, RMSEA = 0.62 (0.057, 0.066), SRMR = 0.08, AIC = 32245.93, BIC = 32664.16]. Life crafting was directly associated with mental health (β = 0.65, *p* < 0.001, *R*^2^ = 0.42) and meaning in life (β = 0.91, *p* < 0.001, *R*^2^ = 0.83). Following this, we examined the structure model which life crafting regressed on job burnout and work engagement in sample 2 (*n* = 362). The model showed adequate fit [χ^2^_(362)_ = 914.29, *df* = 501, χ^2^/*df* = 1.82, CFI = 0.91, TLI = 0.90, RMSEA = 0.48 (0.043, 0.053), SRMR = 0.07, AIC = 26349.81, BIC = 26847.94]. Life crafting was directly associated with job burnout (β = −0.42, *p* < 0.001, *R*^2^ = 0.18) and work engagement (β = 0.54, *p* < 0.001, *R*^2^ = 0.30).

**TABLE 12:** Relationships with burnout, engagement, meaning in life, and mental health.

Regressive path	Standardized					Validity established
	β	SE	*t*-value	*p*	*R* ^2^	
Life crafting→ job burnout	−0.42	0.06	−6.96	<0.01	0.18	Yes
Life crafting→ work engagement	0.54	0.05	10.11	<0.01	0.30	Yes
Life crafting→ mental health	0.65	0.05	13.90	<0.01	0.42	Yes
Life crafting →meaning in life	0.91	0.20	4.47	<0.01	0.83	Yes

## Discussion

The purpose of this paper was to conceptualize life crafting and to develop, validate and evaluate a robust measure of overall life crafting. The results showed that a second-order factorial model of overall life crafting, comprised of three first-order factors called cognitive crafting, seeking social support, and seeking challenges, fitted the data best. Our results, therefore, support the conceptual definition of Life Crafting as the conscious efforts individuals exert to create meaning in their lives through (a) cognitively (re-)framing how they view life, (b) seeking social support systems to manage life challenges and (c) actively seeking challenges to facilitate personal growth. The results further support partial measurement invariance across genders. Moreover, life crafting was substantially different but related to job crafting and proactive personality. Furthermore, life crafting was positively associated with meaning in life, mental health, and work engagement and was negatively related to job burnout. Our results therefore support the notation that life crafting could be a valuable meaning-making strategy which people could employ to create more meaningful life experiences.

### The Life Crafting Framework

The first objective of this paper was to conceptualize and validate a conceptual framework for Life Crafting. Our results support the notion that “Life Crafting” refers to the conscious efforts individuals exert to create meaning in their lives through (a) cognitively (re-)framing how they view life, (b) by seeking social support systems to manage life challenges, and (c) to actively seeking challenges to facilitate personal growth. From this definition, and supported by our empirical findings, Life Crafting is seen to consist out of three factors that provide individuals with the means to both search for new sources of meaning in their lives but also affords the opportunity to (re)craft life in such a way to allow for the self to something larger than themselves:

1. *Cognitive crafting.* Our results indicate that cognitive crafting is an essential component of one’s life crafting strategy. Cognitive crafting is defined as the individual’s ability to proactively reshape or cognitively re-frame the physical, cognitive or social features of work or life in order for it to be perceived as more meaningful.
2. *Seeking social support.* Human beings are fundamentally social animals with a desire to connect to others and its therefore not surprising that seeking social support was found to be a component of life crafting. Seeking social support refers to the extent to which individuals seek out social support systems and networks to achieve personal/professional goals and aid in managing adversity. Meaning is therefore crafted through establishing mutually beneficial relationships with others.
3. *Seeking challenges*. The inherent need to grow and develop ourselves is at the core of most meaning-making strategies (van Zyl et al., 2020). Seeking challenges refers to the active efforts implemented by individuals to stretch their current capabilities and to learn new skills/abilities aimed at facilitating personal growth and environmental mastery.

These factors conceptually overlaps with both the three factors of Wrzesniewski and Dutton’s (2001) conceptualization of job crafting (i.e. cognitive-, relational-, and task crafting) and two of Tims and Bakker (2010)’s conceptualization (i.e. seeking social resources, increasing challenges). This is probably not a surprise as these forms of (job) crafting may lead to the satisfaction of basic human needs, i.e., cognitive crafting (need for positive self-image), relational crafting (need for relatedness), and task crafting (need for competence). In addition, according to Baumeister and Vohs (2002), the essence of the meaning is the need to establish connections with others. When individuals feel that their lives are connected with something larger than themselves and that they are making a significant contribution to society, they intend to appraise their lives as full of meaning (Wong, 2020). When individuals actively seek challenges in their lives, it could lead to either a physical- or perceptive increase in their available resources. Further, we believe that when individuals are able to cognitively craft their lives and are able to seek out the necessary social support needed to facilitate goal achievement, that it could close the gap between one’s current life and desired life. As such, individuals would be able to more actively see how current life tasks relate to their overall goals and therefore result in life feeling more meaningful.

### Psychometric Properties of the Life Crafting Scale

The results further showed that second-order factorial model for overall Life Crafting, comprised out of three first-order factors (cognitive crafting, seeking social support, and seeking challenges) fitted the data best. This was in contrast to the initial expectation that life crafting would be a multi-dimensional construct comprised out of eight factors. Study 1 showed that only three factors could be meaningfully extracted from the data, which was confirmed in Study 2. The reason for such may be threefold. Firstly, the original eight dimensions and their items were primarily derived from other domain-specific studies on crafting behaviors and their associated scales. Given the conceptual overlap between these different crafting approaches, creating an item pool with similarly worded items may have created factors that look rather homogenous to participants. This was in contrast to initial expectations as we expected different approaches to cognitive crafting, for example, to produce different factorial structures. For example, Bindl et al. (2019) measured cognitive crafting with items such as “I thought about ways in which my job as a whole contributed to society” and “I thought about how my job contributed to the organization’s goals.” Whereas Slemp and Vella-Brodrick (2013) measured cognitive crafting with items such as “Think about the ways in which your work positively impacts your life” and “Reflect on the role your job has for your overall wellbeing.” From these two sets of items, it would seem as though the strategies employed to cognitively craft work would consist of two subdimensions: transcending personal goals and positive thinking. In a similar vein, relational crafting could also be conceptually divided into creating new relationships, optimizing current relationships, and utilizing social resources. However, it would seem as though participants do not differentiate between micro-level crafting behaviors but rather just focus on changing the way in which they view life, seeking resources to support their meaning-making processes, and engaging in challenges to stretch their current capabilities in order for them to grow.

Secondly, the results of EFA showed that resources crafting could be removed as a potential factor of life crafting. This dimension was derived from job crafting’s dimension, i*ncreasing structural job resources* (Tims et al., 2012). Increasing structural job resources refers to employees’ proactive behaviors initiated in order to increase resource variety, develop new opportunities, and enhance autonomy at work. However, in the global life domain, there are relatively few situations where people are required to deal with “structure,” which might be why this specific crafting domain did not manifest as initially expected. Further, whilst controlling for environmental factors, one would also assume that individuals are rather autonomous in the way in which they approach life. This is in contrast to work where the roles, functions, processes, and procedures are usually relatively well defined and leave little room for autonomy (Van Zyl et al., 2010). As such, there may be no need to correct for a lack of autonomy through “seeking structural resources” in general life.

Thirdly, demands crafting was also not found to be a component of life crafting. We think that this is because demands crafting is driven by different motivations in contrast to the other factors. In previous studies, demands crafting related to individuals’ avoiding motivation, whereas seeking resources and seeking challenges were connected with approaching motivation (Tims et al., 2012; Lichtenthaler and Fischbach, 2019). Therefore, some researchers found that reduced demands always showed insignificance results with other dimensions (Tims et al., 2012; Lichtenthaler and Fischbach, 2019). Similar to the findings of job crafting, reducing demands was not found to be an essential element of life crafting as it probably does not represent a purposeful behavior contributing to meaning but perhaps occurs due to other reasons and needs, e.g., creating sufficient time and energy for meaningful activities.

Finally, the results showed that the second-order life crafting model demonstrated configural, metric, and partial scalar invariance across gender. These findings imply that overall life crafting, showed similar factor structures, factor loadings, intercepts, and residual errors for both male and female samples. However, seeking social support showed a difference when we tested the scalar invariance. This finding showed that women, on average, seek more social help than men. This result is consistent with other studies showing that women are more inclined than men to seek help when they encounter destructive/difficult/challenging issues (Koydemir-Özden, 2010; Liddon et al., 2018). The potential reason for the difference is that the female participants held more positive attitudes toward help-seeking behaviors and more easily recognized their needs for help than male participants (Ang et al., 2004).

### The Relationship Between Life Crafting and Individual Outcomes

As we expected, life crafting showed a positive relationship with seeking job resources, seeking job challenges, and proactive personality. This result indicated that life crafting might tap the same conceptual area as job crafting and proactive personality. There was no significant relationship between life crafting and a reduction in job demands in the current study. This result implies that life crafting and reducing demands might be two independent variables. One reason for this is that we did not include any demands-based items into the final scale. Another reason may be that reducing job demands is driven by the motivation to avoid, whereas seeking job resources and seeking job challenges have a focus on a more proactive motivation (Hu et al., 2020).

Finally, the current study confirmed the criterion validity of life crafting. We found a positive relationship between life crafting and meaning in life, mental health, and work engagement. These results are consistent with previous studies. For example, Tims et al. (2016) found that crafting behaviors in the work domain could increase meaningfulness. Slemp and Vella-Brodrick (2014) pointed out that job crafting may improve mental health by satisfying personal intrinsic needs. In addition, we found a negative relationship between life crafting and job burnout. This result is in line with Tims et al. (2013) finding that job crafting plays a vital role in preventing job burnout. Overall, the validity of the Life Crafting Scale is further supported by the results and implies that life crafting could be an important predictor for people’s mental condition or state.

### Limitations and Recommendations

Despite the novelty of the current study, it is not without its limitations. First, we only collected cross-sectional data. Therefore, the scale’s stability over time is unknown. According to our definition, life crafting is a self-driven strategy to create meaning, which means life crafting might change over time while the individuals’ motivation changes. Therefore, the longitudinal stability or invariance of the life crafting scale should be investigated in future studies.

Second, all of our criterion indicators relied upon self-report measures. Self-report measures are more open to positive bias, and research has shown that there could be a discrepancy between what is being felt and what is being reported (Van Zyl and Ten Klooster, 2022). This may lead to higher levels of common method effects and positive reporting bias. Future research should aim to validate the Life Crafting Scale against more objective indicators of positive mental health, meaning, and job performance.

Thirdly, the current study checked the measurement invariance of life crafting across gender. However, life crafting may also vary across age and occupations. Future researchers could examine the factorial equivalence of the Life Crafting scale across these different demographic factors. Finally, the cross-cultural validity of the instrument should be investigated. In the current study, the empirical validation of the instrument was only conducted within a western, predominantly individualistic, cultural context. Therefore, future studies should attempt to validate the scale in other cultural contexts such as in eastern countries in order to provide more evidence as to its cross-cultural applicability.

Finally, our approach to life crafting is founded in the philosophical tenents of the meaning-making theory, the conservation of resources theory, and the extension of the Job-Demands and Resources model. Although we believe that our approach is holistic and encompassing, there may be other interpretative frameworks that may also explain life crafting behaviors (e.g., positive existential psychology or chaos theory). For example, Gleick (1988) proposed that we are living in a chaotic world; therefore, our plans are consistently changing because of disruption by external, unplanned events. A typical example is the Covid-19 crisis which hindered our life in various ways yet made us rethink the value and purpose of suffering, the meaning of our lives, and how these affect our mental health or wellbeing (Wong, 2020; Wong et al., 2021). Positive existentialism indicated that the pandemic also resulted in people adopting new strategies to form-, search for or create meaning through reframing the purpose and function of suffering (Wong et al., 2021). These approaches could also be seen or interpreted as life crafting strategies, which reframes how people view essential life events or create meaning from suffering (Wong et al., 2021). Therefore, we urge future researchers to expand upon life crafting theory by approaching such from different philosophical traditions.

## Conclusion

In conclusion, our first attempt to conceptualize and measure life crafting as a global meaning-making strategy has shown promising results. Our results support the importance of life crafting as a tool individuals can employ to enhance their mental health. We also found that crafting behaviors transcends context and that individuals approach meaning-making from a holistic, integrated perspective. Life crafting could therefore be important and alternative strategies researchers and practitioners could use to aid their individuals to find more meaning in their lives.

## Data Availability Statement

The raw data supporting the conclusions of this article will be made available by the authors, without undue reservation.

## Ethics Statement

The studies involving human participants were reviewed and approved by the Ethics Review Committee of Eindhoven University of Technology (under registration number: ERB2020IEIS20). The patients/participants provided their written informed consent to participate in this study.

## Author Contributions

SC collected and analyzed the data and drafted the first version of the manuscript. LM, LZ, and ED made substantial contributions to the drafting of the final manuscript and to the revisions. All authors contributed to the conceptualization and design of the study.

## Conflict of Interest

The authors declare that the research was conducted in the absence of any commercial or financial relationships that could be construed as a potential conflict of interest.

## Publisher’s Note

All claims expressed in this article are solely those of the authors and do not necessarily represent those of their affiliated organizations, or those of the publisher, the editors and the reviewers. Any product that may be evaluated in this article, or claim that may be made by its manufacturer, is not guaranteed or endorsed by the publisher.

## Appendix

**Appendix 1:** The Life Crafting Scale.

Instructions	For the following set of questions, think about how you approach different components of your life. Consider the strategies you employ to create meaningful life experiences. Then rate each item as it applies to your life.					
No	Item	Never	Sometimes	Regularly	Often	Always
***Cognitive crafting***						
1	I think about how my life helps others	1	2	3	4	5
2	I think about how my actions positively impact my community	1	2	3	4	5
3	I think about how my life contributes to society	1	2	3	4	5
***Seeking social support***						
4	I actively ask people for advice when I encounter difficulties	1	2	3	4	5
5	I seek support from my family when I am down	1	2	3	4	5
6	I am willing to ask others for help when things become too difficult to bear	1	2	3	4	5
***Seeking challenges***						
7	I try to work hard on challenging activities	1	2	3	4	5
8	I change my activities so that they are more challenging	1	2	3	4	5
9	I seek out opportunities that challenge my skills and abilities	1	2	3	4	5
**Scoring:**	Create an average or “mean” score of the following items to create a score for each of the components of the Life Crafting Scale					
	1. **Cognitive Crafting** = (Item 1 + Item 2 + Item 3)/3					
	2. **Seeking Social Support** = (Item 4 + Item 5 + Item 6)/3					
	3. **Seeking Challenges** = (Item 7 + Item 8 + Item 9)/3					
	To create an overall score of Life Crafting, create an average score of the means for each of the aforementioned components					
	4. **Overall Life Crafting** = (Cognitive Crafting + Seeking Social Support + Seeking Challenges)/3					
